# Genetic Analysis of Zoonotic Gastrointestinal Protozoa and Microsporidia in Shelter Cats in South Korea

**DOI:** 10.3390/pathogens9110894

**Published:** 2020-10-27

**Authors:** Dongmi Kwak, Min-Goo Seo

**Affiliations:** 1 College of Veterinary Medicine, Kyungpook National University, Daegu 41566, Korea; dmkwak@knu.ac.kr; 2 Cardiovascular Research Institute, Kyungpook National University, Daegu 41944, Korea; 3 Veterinary Drugs and Biologics Division, Animal and Plant Quarantine Agency, Gimcheon 39660, Korea

**Keywords:** cat, *Cryptosporidium felis*, *Giardia duodenalis*, *Blastocystis* sp., *Enterocytozoon bieneusi*, *Toxoplasma gondii*

## Abstract

Feral cats that are roaming outside can serve as reservoirs for zoonotic pathogens, negatively impacting public health. They may experience high levels of parasitic infection. Some gastrointestinal protozoa and microsporidia possessing zoonotic potential in cats include *Cryptosporidium* spp., *Giardia duodenalis*, *Blastocystis* sp., *Enterocytozoon bieneusi*, and *Toxoplasma gondii*. Here, we show the infection rates and risk factors of intestinal protozoa and microsporidia detected from shelter cats on Jeju Island in South Korea in 2020. Among 158 cats, we detected genes for five internal protozoa and microsporidia, namely, *Cryptosporidium felis* (0.6%), *G. duodenalis* (3.8%), *Blastocystis* sp. (0.6%), *E. bieneusi* (3.8%), and *T. gondii* (1.3%). Furthermore, 16 cats (10.1%) were PCR-positive for at least one protozoan or microsporidium. To our knowledge, this study is the first to describe the existence of *C. felis*, *G. duodenalis* assemblage F, *Blastocystis* sp. ST4 subtype, and *E. bieneusi* genotype Peru11 in cats in South Korea. Despite the small number of positive samples, this study expands our understanding of the incidence of zoonotic gastrointestinal protozoa and microsporidia in shelter cats and genetically characterizes the isolates found in the infected animals. Moreover, these findings emphasize the need for a better control strategy on protozoa and microsporidia in cats, tailored to their individual needs, to protect animal and public health.

## 1. Introduction

Cats can act as reservoirs for gastrointestinal pathogens, which may result in public health crises, negative economic results, and poor health in humans and animals [1]. Cats that are roaming outside can pollute the environment with fecal cysts, oocysts, spores, and eggs of pathogens that can infect animals and humans. Intestinal protozoa and microsporidia with zoonotic potential in cats include *Cryptosporidium* spp., *Giardia duodenalis*, *Blastocystis* sp., *Enterocytozoon bieneusi*, and *Toxoplasma gondii*. Humans obtain these pathogens through the intake of contaminated water or food (water- or food-borne transmission), exposure to infected animals (zoonotic transmission), or other humans (anthroponotic transmission) [2]. Some are commensal organisms, whereas others are associated with chronic or acute diarrhea and other gastrointestinal sufferings in the host [3]. For example, *Cryptosporidium* spp. and *G. duodenalis* infections in humans are often completely asymptomatic [4]. Hence, subclinical infections due to these species are also usual in companion animals, including cats. These are therefore important, although frequently ignored, hazards to public health [3].

Universally, the existence of large populations of feral cats have become a highly debated topic due to their impact on public health, the environment, animal welfare, and cat overpopulation, as well as due to discrepancies about the best procedures for their control [5]. Feral cats are significant wild animals because they inhabit the highest position in the urban food-chain and their population has steadily increased. These cats create territory in the same manner as wild animals and usually stay away from each other in each region. Therefore, it is expected that the life cycle of the pathogen is contained within the territory of each cat in each region [6]. This is therefore simultaneously a potential zoonotic risk to animals and people [6].

In South Korea, large populations of stray cats can be seen roaming inhabited streets and have become a target of public complaints. They cause traffic accidents, make noise, and disturb the sleep of humans, which has been a considerably serious topic [6]. Feral cats that are born and live in the wild do not experience the same level of veterinary care and farming as most pet cats, including regular medical checkups and fecal pathogen tests [7]. In addition, these feral cats are most likely to intake pathogens through paratenic hosts or predation of intermediate stage compared to domestic pet cats. Therefore, feral cats may experience a relatively high level of pathogen infection [7]. Available prevalence data for zoonotic pathogens in cats have been highly variable depending on the sample population, regions, and testing methods. The purpose of this study was to examine the presence and infection rates of zoonotic intestinal protozoa and microsporidia in shelter cats in South Korea as well as to assess the risk factors (age and sex) that impact infections and zoonotic potential.

## 2. Results

### 2.1. nPCR and Molecular Identification

In total, 10.1% (16/158, 95% confidence interval (CI): 5.4–14.8) of cats tested PCR-positive for at least one protozoan or microsporidian species in the tested cats (Table 1). The 18S rRNA nucleotide sequences of *Cryptosporidium* sp. (1/158, 0.6%, 95% CI: 0–1.9) was detected. The glutamate dehydrogenase (*gdh*) gene sequences of *G. duodenalis* were detected in 3.8% of the tested cats (6/158, 95% CI: 0.8–6.8). In addition, the *β-giardin* genes of *G. duodenalis* were amplified from these six positive samples. The 18S rRNA nucleotide sequences of *Blastocystis* sp. (1/158, 0.6%, 95% CI: 0–1.9) were also detected. The ribosomal internal transcribed spacer (ITS) region sequences of *E. bieneusi* (6/158, 3.8%, 95% CI: 0.8–6.8) and the B1 gene sequences of *T. gondii* (2/158, 1.3%, 95% CI: 0–3.0) were detected. In this study, multiple infections of protozoa or microsporidia were not observed.

In this study, there were no significant differences in the risk factors of age and sex. However, cats younger than six months of age demonstrated a higher positive rate for intestinal protozoa and microsporidia than those older than six months old, while there was no statistical significance.

### 2.2. Molecular and Phylogenetic Analyses

**Figure 1:**
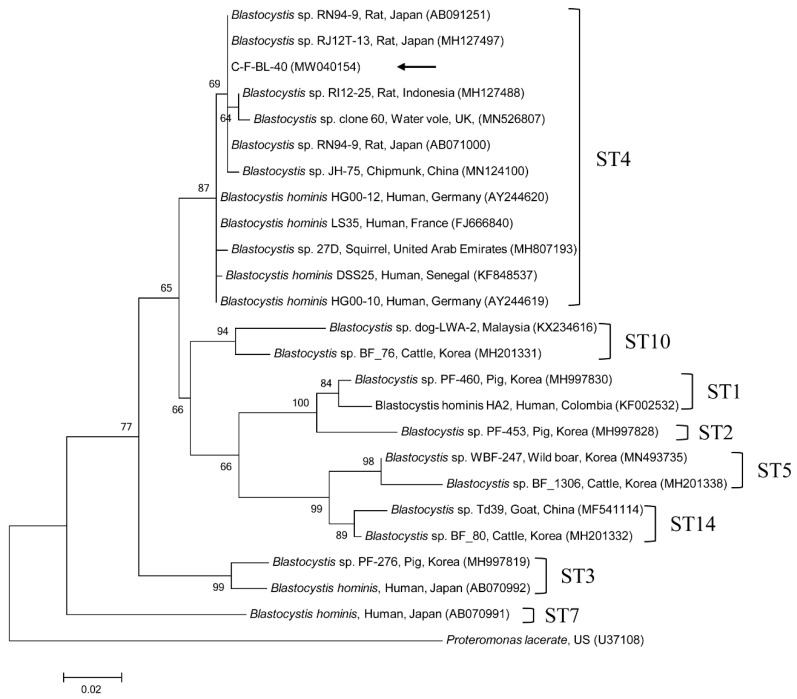
Phylogenetic tree of *Blastocystis* sp. based on 18S rRNA gene sequences. The maximum likelihood method was used to construct the tree, and the arrow indicates the sequence detected in this study. The GenBank accession numbers are shown in parentheses, and the *Blastocystis* subtypes (STs) are indicated. *Proteromonas lacerate* was used as the outgroup. Branch numbers mean bootstrap support levels (1000 replicates) and the scale bar displays the substitution numbers for each nucleotide.

**Figure 2:**
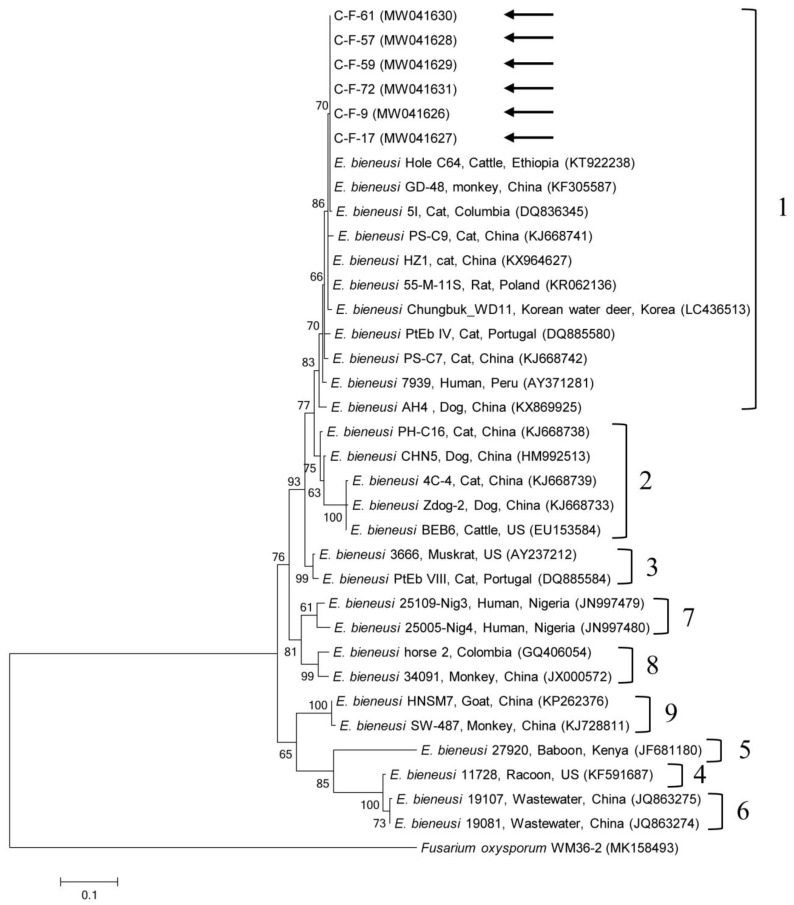
Phylogenetic tree of *Enterocytozoon bieneusi* based on ribosomal internal transcribed spacer (ITS) region sequences. The maximum likelihood method was used to construct the tree and arrows indicate the sequences detected in this study. The GenBank accession numbers are shown in parentheses and the *E. bieneusi* genotypic groups are indicated. *Fusarium oxysporum* was used as the outgroup. Branch numbers mean bootstrap support levels (1000 replicates) and the scale bar displays the substitution numbers for each nucleotide.

Phylogenetic analyses showed that the 18S rRNA nucleotide sequences of *Cryptosporidium* spp. (Supplementary Figure S1), the *gdh* gene (Supplementary Figure S2) and *β-giardin* gene (Supplementary Figure S3) nucleotide sequences of *G. duodenalis*, the 18S rRNA nucleotide sequences of *Blastocystis* sp. (Figure 1), the ITS region (Figure 2) nucleotide sequences of *E. bieneusi*, and B1 gene (Supplementary Figure S4) nucleotide sequences of *T. gondii* were clustered with formerly documented sequences.

One sequence shared 96.8–99.7% identity with the 18S rRNA sequences in formerly reported *C. felis* isolates in GenBank. The six *gdh* gene sequences of *G. duodenalis* found in this study shared 100% identity with each other. These six sequences also shared 99.7–100% identity with the *gdh* gene sequences in formerly reported *G. duodenalis* assemblage F isolates in GenBank. The six *β-giardin* gene sequences of *G. duodenalis* identified in this study shared 100% identity with each other and 99.9–100% identity with the *β-giardin* nucleotide sequences in formerly reported *G. duodenalis* assemblage F isolates in GenBank. Another sequence shared 99.4–100% identity with the 18S rRNA sequences in formerly reported *Blastocystis* sp. subtype (ST) 4 isolates in GenBank. The six ITS region sequences of *E. bieneusi* found in this study shared 100% identity with each other and 98.8–100% identity with the ITS region sequences in the formerly reported *E. bieneusi* group 1 isolates in GenBank. The two B1 gene sequences of *T. gondii* found in this study shared 100% identity with each other. These two sequences also shared 96.1–100% identity with the B1 gene sequences in formerly reported *T. gondii* isolates in GenBank.

The representative sequences reported in the present study were submitted to GenBank, with the following accession numbers: MW040153 (*C. felis* 18S rRNA), MW048644–MW048649 (*G. duodenalis gdh* gene), MW048638–MW048643 (*G. duodenalis β-giardin* gene), MW040154 (*Blastocystis* sp. 18S rRNA), MW041626–MW041631 (*E. bieneusi* ITS region), and MW063447–MW063448 (*T. gondii* B1 gene).

## 3. Discussion

This study showed that 10.1% of shelter cats were infected with at least one species of intestinal protozoa or microsporidia, and infection rates in the shelter cats in this study were lower than expected as per previous studies [6,8]. In a previous study, shelter cats had the highest infection rates—55.9% were infected with at least one species of an intestinal pathogen compared to 33.2% of the household cats [8]. In general, sheltered animals harbor higher prevalence of intestinal pathogens due to crowded conditions and poor hygienic situations. We only surveyed a small number of samples in the restricted region of Jeju Island in South Korea. These limitations may have affected the infection rates of gastrointestinal protozoa and microsporidia in this study. Therefore, large scale surveys are required in more regions of South Korea, such as the mainland. Despite the high prevalence of protozoan and microsporidian infections, most animals were healthy with no clear signs of symptoms, possibly due to the low parasitic load, as indicated by the low number of oocyst/cyst/egg output reported in most cases [8]. Though there was not a clear association between clinical signs and numbers of oocysts/cysts/eggs [8], in this study, young cats demonstrated a higher positive rate for intestinal protozoa and microsporidia than old cats, but this was not statistically significant. Normally, young animals are more sensitive to parasitism [9], and other studies have confirmed that older animals tend to be less infected [8].

In the present study, *Cryptosporidium* spp., *G. duodenalis*, *Blastocystis* sp., *E. bieneusi*, and *T. gondii* were detected in cats through molecular analysis. *Cryptosporidium* spp. are the major causative agents of zoonotic diarrhea globally [3]. *Cryptosporidium* oocysts are directly infectious upon defecation, remain in the environment for many months, are resistant to chlorine medication, and may be excreted in large numbers by an infected individual host [10]. Recently, at least 38 species of *Cryptosporidium* have been recognized, and most of these are host-adapted [11]. *Cryptosporidium parvum* and *Cryptosporidium hominis* are zoonotic species, and *C. felis* is the major prevalent species in cat, although *Cryptosporidium ryanae*, *C. parvum*, *Cryptosporidium muris, Cryptosporidium* rat genotype III, and a novel genotype most closely related to *Cryptosporidium* rat genotype III have also been detected in cats [12]. In South Korea, *Cryptosporidium* spp. have been reported in fecal samples from cattle (9.9%, 94/951), including *Cryptosporidium bovis/C. ryanae* and *C. parvum* [13]; nevertheless, there was no report about cat infections. Furthermore, there are numerous studies that have detected *C. felis* in cats worldwide, such as in fecal samples in China (2.3%, 8/346; 3.8%, 6/160) [2,14] and Greece (6.8%, 18/264) [8], and *Cryptosporidium* spp. in the US (3.3%, 6/180) [6]. In our study, only one cat tested positive for *C. felis* by 18S rRNA gene analysis. To our knowledge, this is the first report on the presence of *C. felis* in cats in South Korea. The risk of human cryptosporidiosis caused by *C. felis* is believed to be quite low, and most of the confirmed cases have been detected in immunocompromised patients because *C. felis* infection can be transmitted from cats to humans [15]. In a previous study, pet animals were not considered as natural reservoirs of human cryptosporidiosis with respect to *C. felis* in cats and *Cryptosporidium canis* in dogs [16]. Thus, zoonotic transmission of cryptosporidiosis between pet animals and their owners should be regarded as a rare event that can only occur in specific conditions [16].

*G. duodenalis* (also known as *Giardia lamblia* and *Giardia intestinalis*) is the only human infective *Giardia* species that can also infect many other mammals, including cats [17]. *Giardia* cysts survive for many months in the environment, and intake of as few as 10 cysts can result in severe dehydration, weight loss, and diarrhea in people and animals [18]. This species includes eight different genetic assemblages (A–H) based on recent molecular genetic features and host range [17]. This includes the zoonotic assemblages A and B, the host-adapted assemblages C and D in dogs, assemblage E in pigs and ruminants, assemblage F in cats, assemblage G in rats and mice, and assemblage H in aquatic mammals [2]. In South Korea, *G. duodenalis* has been detected in fecal samples from dogs (15.5%, 99/640) [19] and pigs (14.8%, 110/745) [20], but there are no reports about cat infection. There are several international studies about cat infections based on analysis of fecal samples in China (13.1%, 21/160; 1.4%, 5/346) [2,14], Greece (20.5%, 54/264) [8], and the US (5.7%, 11/192) [7]. Assemblage F is most prevalent in cats, but infrequent infections with zoonotic assemblages A and B have also been reported in cats [2,8,14]. In our study, six cats (3.8%) tested positive for *G. duodenalis*, and assemblage F was identified through *gdh* and *β-giardin* gene expression. In other studies, infected cats also presented with assemblages A–D and F [2,8,14]. Furthermore, another study confirmed that *G. duodenalis*-positive samples were more sensitive to small subunit (*ssu*) rRNA compared with the protein-coding genes *gdh* and *β-giardin* [20]. For detection purposes, *ssu* rRNA is preferred because it is a multi-copy, highly sensitive gene, whereas protein-coding genes exhibit low sensitivity because they are single-copy genes that are typically used for genotyping purposes [21]. However, several studies utilized multilocus methods, including *gdh* and *β-giardin* for genotyping [19,20]. Unfortunately, we evaluated only *gdh* and *β-giardin* genes in this study; therefore, the actual prevalence of *G. duodenalis* in the cat population surveyed here may be underestimated. To our knowledge, this is the first report on the presence of *G. duodenalis* in cats in South Korea. However, zoonotic genotype assemblages A and B were not detected in the present study. Based on previous studies, the entire impact of zoonotic transmission from pet animals, such as assemblage C in dogs and assemblage F in cats, to/from humans are perhaps insignificant, but cannot be normally excepted [16,22]. 

*Blastocystis* sp., a single-celled pathogen belonging to stramenopiles, is frequently detected in fecal samples from animals and humans [3]. Although the pathogenic role of *Blastocystis* sp. remains controversial, emerging clinical, laboratory, and epidemiological evidence suggests a relationship between extra-intestinal (urticarial) and gastrointestinal (diarrhea and irritable bowel syndrome) disorders [23], and another study considered *Blastocystis* sp. as a commensal that is part of the healthy gut flora [24]. Indeed, *Blastocystis* is common in asymptomatic, apparently healthy individuals globally. For instance, *Blastocystis* sp. infections in humans are often completely asymptomatic [4]. Wide intragenetic variations among *Blastocystis* sp. isolates have been reported, leading to the identification of 22 taxonomically valid distinct STs. The STs associated with infections in humans and animals are 1–9 and 12, whereas STs 10, 11, 13–17, 21, and 23–26 have only been detected in animals [25]. *Blastocystis* sp. is an unusually successful pathogen with a huge array of host species. In humans, nine STs of *Blastocystis* have been reported (ST1–9). The most prevalent STs are ST1–4, and the major type is ST3, which is present in about 60% of cases [26]. Moreover, non-human specific STs have been reported in other animals. In South Korea, *Blastocystis* has been detected in fecal samples from cattle (6.7%, 101/1512) [27], wild boars (10.4%, 45/433) [28], and humans (9.0%, 29/324) [29], but there is no report describing cat infection. However, there are a few studies about cat infections globally, such as in fecal samples in China (0.6%, 2/346) [14], Australia (100%, 3/3) [30], and the US (11.4%, 12/105) [31]. In our study, only one cat tested positive for *Blastocystis* sp., identified as ST4 type by 18S rRNA gene analysis. In other studies, infected cats also presented with ST1, 3, 4, and 10 [14,30,31]. ST4 is generally believed to be a rodent-adapted subtype [32,33]. Although more information is obviously needed to confirm the host specificity of ST4, the ST4 strains are shown to be dominantly preserved in wild rodents; and the potent infections of ST4 to other host species, such as kangaroo, monkey, and human, are regarded to be accidental [32]. In another study, an ST4 strain was first detected in symptomatic patients and, subsequently, closely contacted households, cats, and dogs were also infected with ST4 [30]. Hence, if cats harbor ST4, it is plausible that they may have acquired after ingesting an infected rodent or transmitted by other hosts, such as humans. Interestingly, shelter-resident companion animals are at higher risk for harboring *Blastocystis* sp., whereas there was no infection in client-owned companion animals [30]. This phenomenon was also shown in shelter cats tested in the present study. To our knowledge, this is the first report on the presence of *Blastocystis* sp. in cats in South Korea. There is generally a high infection rate of STs in animal handlers, and these STs have also been detected in the feces of animals in contact with handlers [34]. However, pet dogs and cats play a potentially insignificant role as natural reservoirs of human *Blastocystis*, although the applicability of these results should be confirmed in further molecular epidemiological researches [35].

A unicellular microsporidium, *E. bieneusi*, affects a widespread range of mammalian hosts, including humans, globally [36], with >500 genotypes at the ITS locus distributed in some genetically isolated groups based on phylogenetic analysis (zoonotic groups 1–2 and mostly host-adapted groups 3–11) [37]. Furthermore, *E. bieneusi* has been broadly detected in a wide range of animal hosts, including domestic and wild animals, greatly proposing the zoonotic potential of this pathogen in which animals may act as natural reservoirs of human infections [36]. In South Korea, *E. bieneusi* has been detected in fecal samples from wildlife (45.2%, 71/157) [38] and in fecal (1.9%, 4/210) and intestinal tissue (5.2%, 3/58) samples from bats [39]. However, there have been no reports about cat infection. There are some studies on cat infections globally, such as in China (5.6%, 9/160) [2] and Spain (3%, 3/99) [40]. In our study, six cats tested positive for *E. bieneusi* and we detected the Peru11 genotype, which belongs to group 1 via the ITS region. In another study, an infection in cats also demonstrated the existence of the Peru11 genotype [40]. To our knowledge, this is the first report on the presence of *E. bieneusi* in cats in South Korea. This genotype is known to have a broad host spectrum and has been detected in humans, poultry, non-human primates, companion animals, livestock, and wildlife [41]. Identification of this genotype with zoonotic potential represents a public health concern, which warrants additional study in the future.

Toxoplasmosis is a zoonotic disease caused by the protozoon *T. gondii*. Most warm-blooded animals, including humans, can serve as the intermediate host, and oocysts of *T. gondii* are excreted only from the definitive host, which is the family Felidae, including cats [42]. Based on the virulence levels of *Toxoplasma* strains in outbred mice, strains were divided into three genotypes, including types I (highly virulent strain), II (less virulent strain), and III (avirulent strain) [43]. In South Korea, *T. gondii* has been detected in fecal samples from feral cats (0.9%, 5/563) [44] and stray cats (4.7%, 14/300) [45], blood samples from stray cats (38.9%, 28/72) [6], and blood, fecal, and tissue samples from cats (0.2%, 10/4432) [46]. Seroprevalence of *T. gondii* infection was reported in cat sitters (6.4%, 43/673) and people (8.0%, 89/1114) in South Korea [47]. In our study, two cats tested positive for *T. gondii* and we identified type I/III by B1 gene. Further phylogenetic analyses are needed to distinguish between types I and III more clearly. In other studies, infected cats also exhibited types I, II, and III in South Korea [45,46]. Since intake of oocyst-contaminated water or food is the only effective way to get infected with *T. gondii*, the low prevalence of cats with *T. gondii* oocysts in South Korea may decrease the worry and public awareness of cats as the main cause for human transmission of toxoplasmosis [44]. In this manner, appropriate management of feral cats should be ranked as a factor toward better public health.

In this study, although we surveyed five different intestinal protozoa and microsporidia from cats, the limitations of the study included the small number of genotyped isolates for each pathogen and limited sensitivity of *gdh* and *β-giardin* for detecting *G. duodenalis*, among other pathogens. The Korean peninsula is gradually shifting toward a subtropical climate, and Jeju Island is particularly susceptible to this change, owing to its lower latitude. Additionally, Jeju Island is located at the southernmost end of South Korea, with a warm oceanic climate. Temperature and humidity due to the rainfall rate in South Korea are promising environmental factors that maintain the viability of oocysts/cysts/spores/eggs of protozoa and microsporidia long-term and may be accountable for the infection rate [38]. Furthermore, visitors that travel to Jeju Island and abandon their pet animals have an increased risk of exposure to many pathogens, leading to disease transmission to animals and serious impact on public health. Although the infection rates of shelter cats in this study were not as high as we expected, shelter cats are usually sources of pathogens that may cause poor health results in people and animals. Therefore, the best health results hinge on preventing exposure to pathogens. Prevention can be reached through the interdisciplinary relationship of policy makers and human health, veterinary, and public health specialists. Prevention measures should stress public education regarding the risks of zoonotic pathogens related to free-roaming cats as well as efforts to gently decrease feral cat populations. This study was imperfect by the irregular collection of samples from animal shelters in Jeju Island, which may lead to biases in the results. Therefore, a large-scale study covering further geographical areas of mainland South Korea with even sample collection is ongoing to discover the role of cats as a reservoir for zoonotic gastrointestinal pathogens.

## 4. Materials and Methods

### 4.1. Sample Size and Collection

The statistical sample size was decided using a formula with an expected disease prevalence of 10%, a confidence level of 95%, and an accepted absolute error of 5%, with a simple random sampling strategy [48].

Based on the formula, a minimum of 138 samples were required. In this study, we collected fecal samples from 158 cats from shelter residences in Jeju Island in 2020. Data on age and sex were documented for each fecal sample obtained.

### 4.2. DNA Extraction and PCR

Genomic DNA was extracted from fecal samples using QIAamp Fast DNA Stool Mini Kit (Qiagen, Melbourne, Australia), as per the given protocol. AccuPower HotStart PCR Premix Kit (Bioneer, Daejeon, Korea) was employed for PCR amplification. DNA template, primers, and distilled water were added to make a total 20 μL reaction volume (distilled water 16 μL, primers 1 μL each, and DNA 2 μL). A 1.5% agarose solution stained with ethidium bromide was prepared, and PCR products were then loaded in the electrophoresis gel and run at 100 V for 30 min. All results were photographed using a UV transilluminator.

To detect *Cryptosporidium* spp., the 18S rRNA gene was amplified. A fragment of approximately 295 bp was amplified using the 18SiF and 18SiR primers [49]. Each DNA preparation of *G. duodenalis* was characterized using two distinct protocols for nested PCR depending on the target to be amplified. A fragment of about 530 bp of the *gdh* gene was amplified using external (Gdh1 and Gdh2) and internal (Gdh3 and Gdh4) primers [50]. Positive samples were then re-tested using primers designed from the *β-giardin* gene, in which a fragment of approximately 384 bp was amplified using external (G7 and G759) and internal (G376 and G759) primers [51]. To detect *Blastocystis* sp., the 18S rRNA gene was amplified. A fragment of approximately 600 bp was amplified using the RD5 and BhRDr primers [52]. To detect *E. bieneusi*, the ITS region was amplified. A fragment of approximately 390 bp was amplified using external (EBITS3 and EBITS4) and internal (EBITS1 and EBITS2.4) primers [53]. To detect *T. gondii*, the *B1* gene was amplified. A fragment of approximately 531 bp was amplified using external (Tg1 and Tg2) and internal (Tg3 and Tg4) primers [54].

All primers and PCR conditions used for detecting intestinal protozoa or microsporidia from cats in the present study are described in Supplementary Table S1.

### 4.3. DNA Cloning

The purification of amplified gene fragments was performed using QIAquick Gel Extraction Kit (Qiagen), and then inserted into the pDrive vector (Promega, Madison, WI, USA) following the instructions given by the manufacturer. The resulting constructs were then used to transform *Escherichia coli* DH5α competent cells (Thermo Fisher Scientific). The bacteria were incubated at 37 °C overnight, and plasmids were purified using Plasmid Miniprep Kit (Qiagen), following the given instructions.

### 4.4. DNA Sequencing and Phylogenetic Analysis

Purified PCR products obtained using forward and reverse PCR primers were sequenced by Macrogen (Seoul, South Korea), and each raw chromatogram was visually inspected for double peaks using CLC Main Workbench 6.7.2 (CLC Bio, Qiagen, Aarhus, Denmark). Next, sequences were analyzed using the multiple sequence alignment program CLUSTAL Omega (version 1.2.1). Results of sequence alignments were modified using BioEdit (version 7.2.5), and phylogenetic analysis was performed with MEGA (version 6.0) using the maximum likelihood method. The aligned sequences were analyzed with a similarity matrix. The phylogenetic tree stability was assessed by bootstrap analysis with 1000 replicates.

### 4.5. Statistical Analysis

A statistical analysis was performed with the analytical software package GraphPad Prism version 5.04 (GraphPad Software Inc., La Jolla, CA, USA). Fisher’s exact test was used to analyze 2 × 2 tables. A 95% CI was calculated for all estimates.

## Figures and Tables

**Table 1:** Prevalence of zoonotic gastrointestinal protozoa and microsporidia in cats in South Korea in 2020.

Category		No. Tested	*Cryptosporidium* *felis*		*Giardia* *duodenalis*		*Blastocystis* sp.		*Enterocytozoon* *bieneusi*		*Toxoplasma* *gondii*		Total
			Positive (%)	95% CI	Positive (%)	95% CI	Positive (%)	95% CI	Positive (%)	95% CI	Positive (%)	95% CI	
Sex	Female	96	1 (1.0)	0–3.1	3 (3.1)	0–6.6	1 (1.0)	0–3.1	4 (4.2)	0.2–8.2	1 (1.0)	0–3.1	10 (10.4)
	Male	62	0	0	3 (4.8)	0–10.2	0	0	2 (3.2)	0–7.6	1 (1.6)	0–4.8	6 (9.7)
Age (months)	≤6	50	1 (2.0)	0–5.9	3 (6.0)	0–12.6	1 (2.0)	0–5.9	2 (4.0)	0–9.4	1 (2.0)	0–5.9	8 (16.0)
	>6	108	0	0	3 (2.8)	0–5.9	0	0	4 (3.7)	0.1–7.3	1 (0.9)	0–2.7	8 (7.4)
Total		158	1 (0.6)	0–1.9	6 (3.8)	0.8–6.8	1 (0.6)	0–1.9	2 (1.3)	0.8–6.8	2 (1.3)	0–3.0	16 (10.1)

95% CI, 95% confidence interval.

## Supplementary Materials

The followings are available online at https://www.mdpi.com/2076-0817/9/11/894/s1, Table S1: Primers used for detecting internal protozoa and microsporidia from cats in the present study, Figure S1: Phylogenetic tree of *Cryptosporidium* spp. based on the 18S rRNA gene sequences, Figure S2: Phylogenetic tree of *Giardia duodenalis* based on the *gdh* gene sequences, Figure S3: Phylogenetic tree of *Giardia duodenalis* based on the *β-giardin* gene sequences, Figure S4: Phylogenetic tree of *Toxoplasma gondii* based on the B1 gene sequences.
