# Supplementary material for: Genetic Analysis of Zoonotic Gastrointestinal Protozoa and Microsporidia in Shelter Cats in South Korea
Source: Pathogens. 2020 Oct 27;9(11):894. doi: 10.3390/pathogens9110894 (PMC7693795; doi:10.3390/pathogens9110894)
Supplement: Supplementary file 1 [file pathogens-09-00894-s001.pdf]

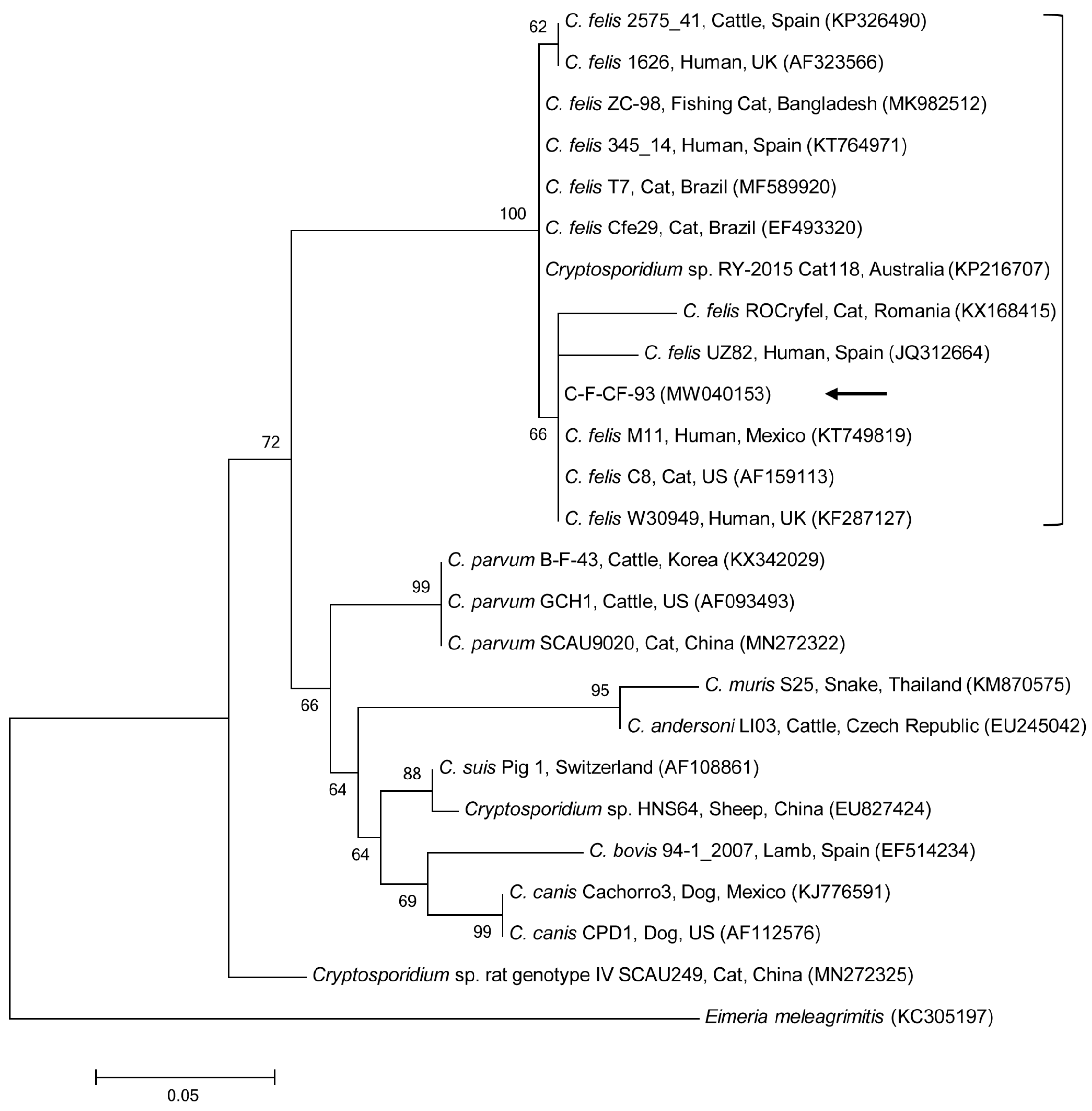

Figure S1: Phylogenetic tree of *Cryptosporidium* spp. based on 18S rRNA gene sequences. The maximum likelihood method was used to construct the tree and the arrow indicates the sequence detected in this study. The GenBank accession numbers are shown in parentheses. *Eimeria meleagrimitis* was used as the outgroup. Branch numbers mean bootstrap support levels (1000 replicates), and the scale bar displays the substitution numbers for each nucleotide.

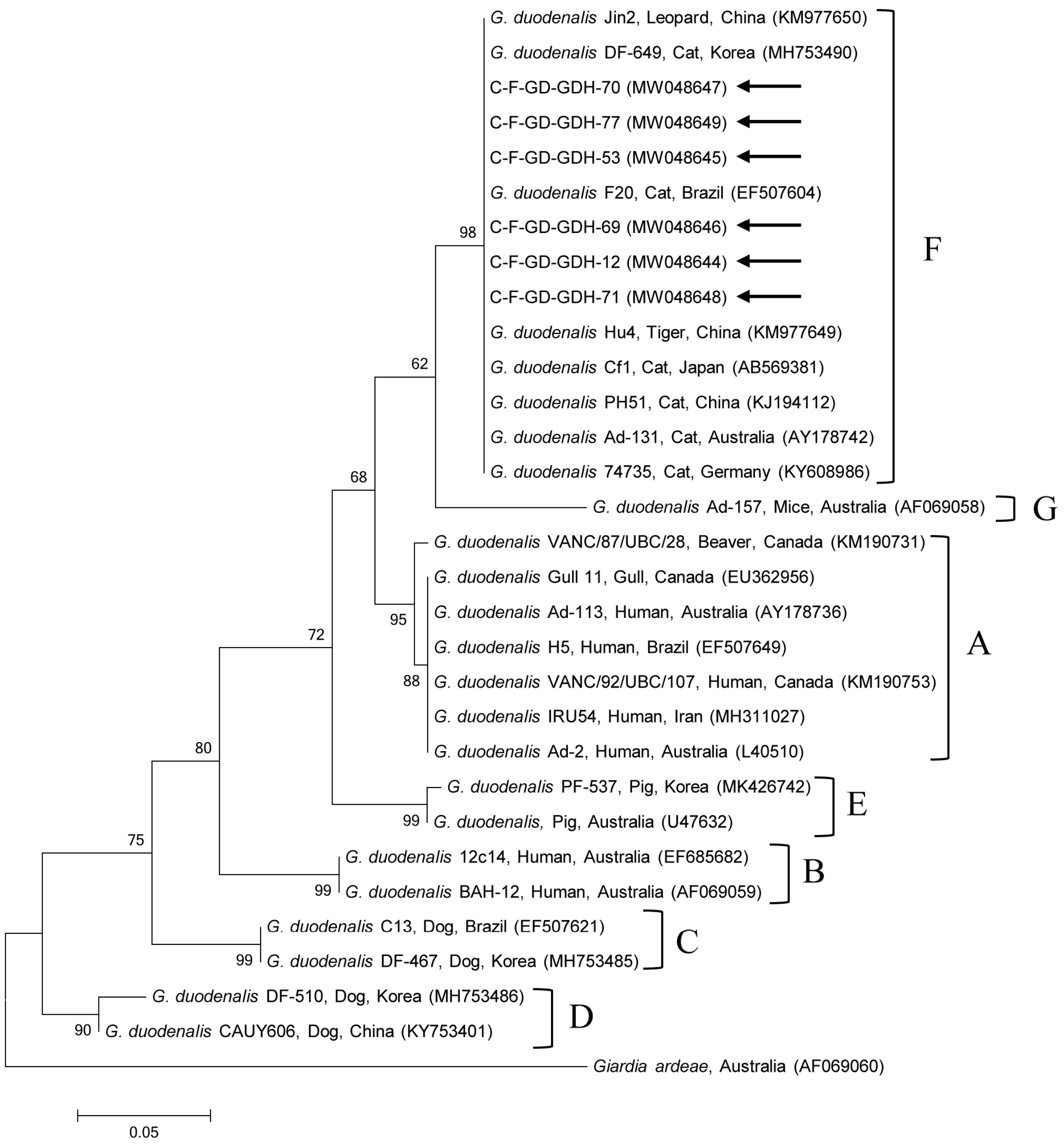

Figure S2: Phylogenetic tree of *Giardia duodenalis* based on the *gdh* gene sequences. The maximum likelihood method was used to construct the tree, and the arrows indicate the sequences detected in this study. The GenBank accession numbers are shown in parentheses and the *G. duodenalis* assemblages A–G are indicated. *Giardia ardeae* was used as the outgroup. Branch numbers mean bootstrap support levels (1000 replicates), and the scale bar displays the substitution numbers for each nucleotide.

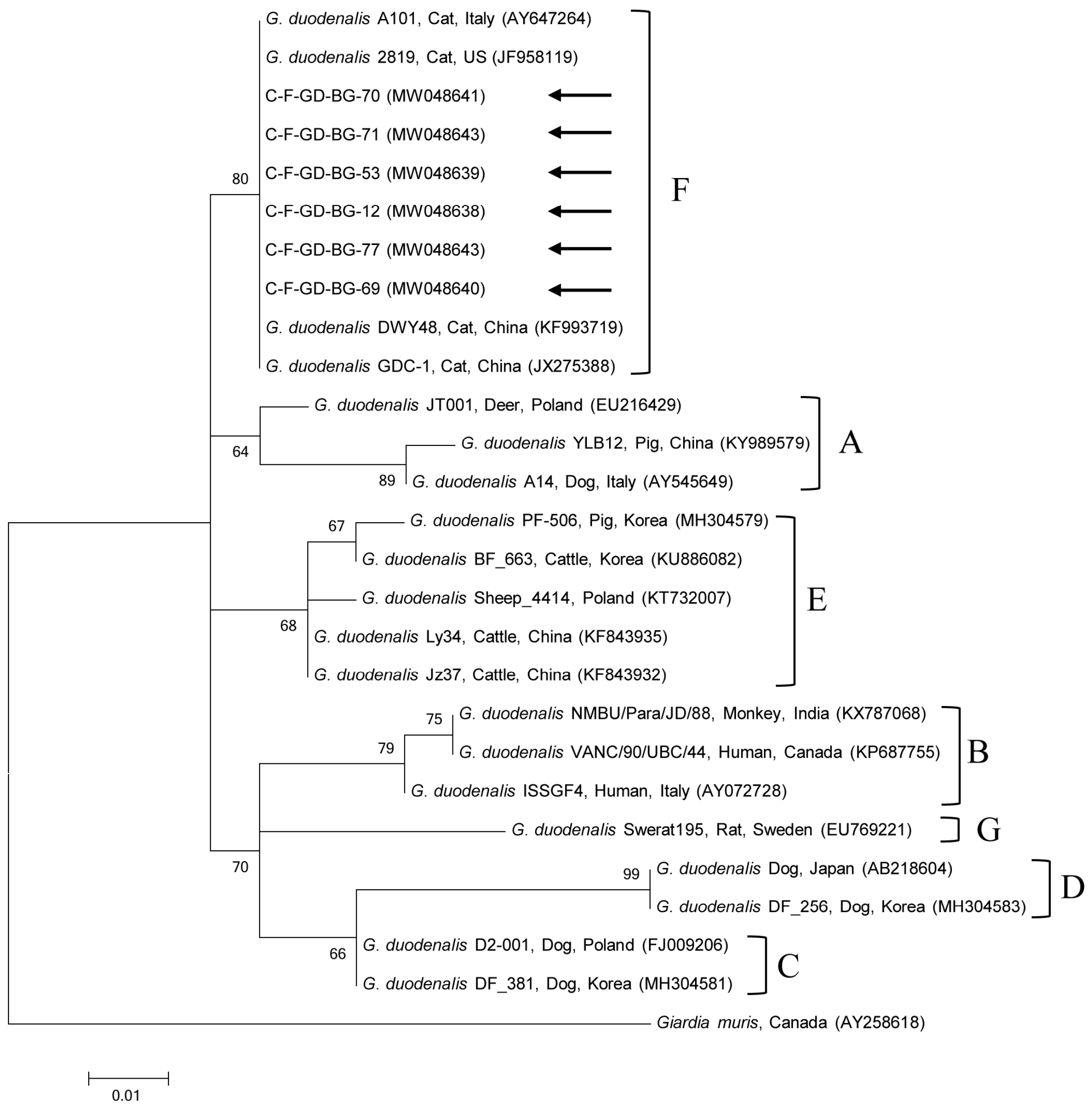

Figure S3: Phylogenetic tree of *Giardia duodenalis* based on *β-giardin* gene sequences. The maximum likelihood method was used to construct the tree, and the arrows indicate the sequences detected in this study. The GenBank accession numbers are shown in parentheses and the *G. duodenalis* assemblages A–G are indicated. *Giardia muris* was used as the outgroup. Branch numbers mean bootstrap support levels (1000 replicates), and the scale bar displays the substitution numbers for each nucleotide.

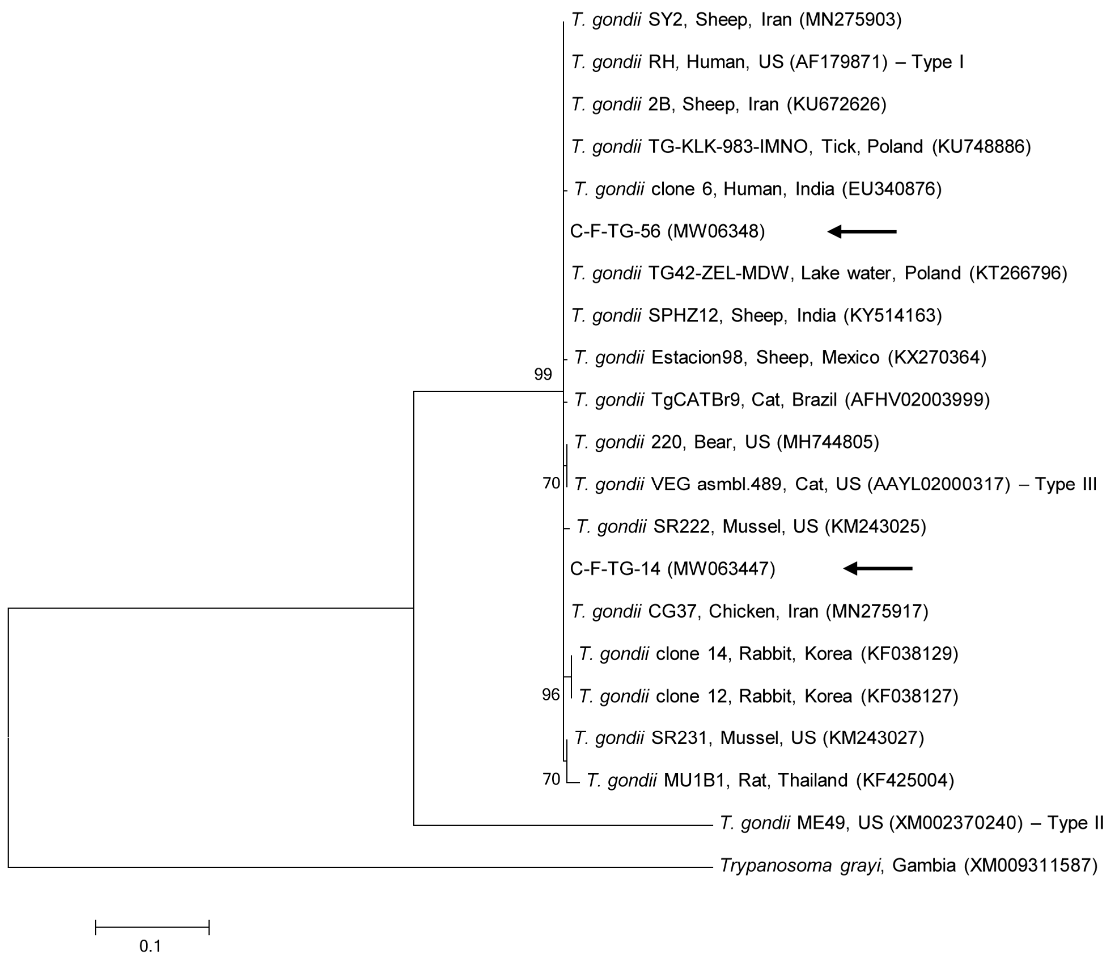

Figure S4: Phylogenetic tree of *Toxoplasma gondii* based on B1 gene sequences. The maximum likelihood method was used to construct the tree, and the arrows indicate the sequences detected in this study. The GenBank accession numbers are shown in parentheses. *Trypanosoma grayi* was used as the outgroup. Branch numbers mean bootstrap support levels (1000 replicates), and the scale bar displays the substitution numbers for each nucleotide.

Table S1: Primers used for the detection of zoonotic gastrointestinal protozoa and microsporidia in cats in the present study.

Organism	Gene	Primer	Sequence 5′ to 3′	Size (bp)	Amplification condition	Reference
Cryptosporidium spp.	18S rRNA	18SiF	AGT GAC AAG AAA TAA CAA TAC AGG	295	94 °C/ 5 min; 35 cycles: 94 °C/ 30 s, 60 °C/ 30 s, 72 °C/ 60 s; 72 °C/ 5 min	Cheun et al., 2007
		18SiR	CT GCT TTA AGC ACT CTA ATT TTC			
Giardia duodenalis	*gdh*	Gdh1	TTC CGT RTY CAG TAC AAC TC	755	94 °C/5 min; 35 cycles: 94 °C/ 30 s, 50 °C/ 30 s, 72°C/ 60 s; 72°C/ 5 min	Cacciò et al., 2008
		Gdh2	ACC TCG TTC TGR GTG GCG CA			
		Gdh3	ATG ACY GAG CTY CAG AGG CAC GT	530		
		Gdh4	GTG GCG CAR GGC ATG ATG CA			

Giardia duodenalis	*β-giardin*	G7	AAG CCC GAC GAC CTC ACC CGC AGT GC	753	94 °C/ 5 min; 40 cycles: 94 °C/ 30 s, 65 °C/ 30 s, 72 °C/ 60 s; 72 °C/ 5 min	Cacciò et al., 2002
		G759	AGG CCG CCC TGG ATC TTC GAG ACG AC			
		G376	CAT AAC GAC GCC ATC GCG GCT CTC AGG AA	384		
		G759	AGG CCG CCC TGG ATC TTC GAG ACG AC			
Blastocystis sp.	18S rRNA	RD5	ATC TGG TTG ATC CTG CCA GT	600	94 °C/ 5 min; 35 cycles: 94 °C/ 30 s, 58 °C/ 30 s, 72 °C/ 30 s; 72 °C/ 5 min	Ramírez et al., 2014
		BhRDr	GAG CTT TTT AAC TGC AAC AAC G			
Enterocytozoon bieneusi	ITS region	EBITS3	GGT CAT AGG GAT GAA GAG	435	94 °C/ 5 min; 35 cycles: 94 °C/ 30 s, 57 °C/ 30 s, 72 °C/ 40 s; 72 °C/ 5 min	Sulaiman et al., 2003
		EBITS4	TTC GAG TTC TTT CGC GCT C			
		EBITS1	GCT CTG AAT ATC TAT GGC T	390	94 °C/ 5 min; 30 cycles: 94 °C/ 30 s, 55 °C/ 30 s, 72 °C/ 30 s; 72 °C/ 5 min	
		EBITS2.4	ATC GCC GAC GGA TCC AAG TG			
T. gondii	B1	Tg1	GTT CTG TCC TAT CGC AAC G	579	94 °C/ 5 min; 35 cycles: 94 °C/ 30 s, 48 °C/ 40 s, 72 °C/ 45 s; 72 °C/ 5 min	Grigg et al., 2001
		Tg2	ACG GAT GCA GTT CCT TTC TG			
		Tg3	TCT TCC CAG ACG TGG ATT TC	516	94 °C/ 5 min; 35 cycles: 94 °C/ 45 s, 56 °C/ 60 s, 72 °C/ 90 s; 72 °C/ 5 min	
		Tg4	CTC GAC AAT ACG CTG CTT GA			
